# Ending the pandemic: reducing new HIV infections to zero

**DOI:** 10.7448/IAS.16.1.18933

**Published:** 2013-12-01

**Authors:** Iryna Zablotska

**Affiliations:** The Kirby Institute, The University of New South Wales, Sydney, Australia

In 2010, the fourth decade of the HIV pandemic arrived during a time of unprecedented success in HIV prevention. Globally, UNAIDS estimated that new HIV infections fell by 33% between 2001 and 2011; new infections among adults and adolescents fell by 50% or more in 26 countries (more than half of these countries were in sub-Saharan Africa), and new infections among children worldwide dropped by 52% [1,2]. The declines in new HIV infections are particularly evident in countries with sustained and more strategic investments, which take into account the specifics of local epidemics, increased political leadership and community engagement in response to the HIV epidemic, and scale up of HIV prevention and treatment programmes [2]. The rapidly growing delivery of antiretrovirals to women and infant feeding–based prevention programmes has resulted in a sharp decline in new HIV diagnoses among children. The encouraging declines in HIV infections can also be attributed to the improved effectiveness of combination antiretroviral treatment (cART), an expanded range of improved medications, the declining prices that make cART more accessible to people in low-income countries, growing coverage with HIV testing, and improved access to prevention and treatment services (particularly for women and young people in low-income countries). Global investment in the AIDS response jumped from US$3.8 billion in 2002 to US$18.9 billion in 2012.

The new decade also saw a revolution in HIV prevention with ground-breaking scientific advances in HIV biomedical prevention, and specifically, proof that microbicides containing an antiretroviral agent can reduce sexual transmission of HIV to women by 39% [3], that earlier start of treatment by HIV-positive people (treatment as prevention or TasP) can reduce the risk of onward transmission by as much as 96% [4], and that consistent, correct use of a daily antiretroviral tablet by men who have sex with men (MSM) can achieve substantial reductions in HIV infections (pre-exposure prophylaxis or PrEP) [5].

In response to the excitement and optimism surrounding the preventative effects of antiretroviral medications, the UN member states considered and unanimously approved the new Political Declaration on HIV/AIDS at a special session of the General Assembly in New York in 2011 [6]. At the core of the 2011 UN Political Declaration are ambitious new HIV prevention targets calling on governments to commit to reducing sexual transmission of HIV by 50%, reducing HIV transmission though injecting drug use by 50% and eliminating mother-to-child transmission of HIV by 2015. These targets are aimed at reinvigorating the commitment towards achieving the Millennium Development Goal #6 to combat HIV/AIDS [7].

In the past two years, research on HIV biomedical prevention has focused on adapting the new prevention strategies to the context of local HIV epidemics [8]. There have been equal measures of optimism and pessimism expressed about the ability of new prevention strategies to halt the HIV pandemic. Based on the evidence that starting treatment earlier can increase health benefits and extend life for people with HIV [9,10], many clinicians are already recommending early treatment for both medical and TasP purposes. Regarding PrEP, the widespread reaction is caution in recommending this strategy. Such reluctance is based on concerns about the common adherence issues in the studies of PrEP [11], the obvious relationship between level of adherence to daily medication schedule and its preventative effect [12] and side effects and drug-resistant HIV [13], among others. Despite little evidence that PrEP use can affect behaviour, many concerns have been voiced about the future of safe sex practices, particularly condom use among MSM, if new biomedical prevention strategies are introduced. More research is needed to investigate this issue using appropriate study designs. In its current form, daily PrEP may benefit only a small number of people with very high and ongoing risk for HIV infection, and other PrEP regimens must be explored. The overarching concern about new prevention strategies, particularly PrEP, is that the cost and burden of providing them are currently unacceptable for most, even high-income, countries. As a result, there have been calls for more evidence and a very slow progress in implementing these two new exciting HIV prevention developments. Regarding PrEP, only two countries to date have prescription guidelines for people at high risk of HIV infection [14,15].

We now have the knowledge and new tools to revolutionize HIV prevention, and we have the bold new Political Declaration with ambitious targets. It must be acknowledged that the task of bringing HIV infections down to zero seems daunting from where we stand now in late 2013. Despite the global success in lowering the HIV infection rates by 33% [1], sub-Saharan Africa has seen only a 25% decline [16]. Some regions have seen increases (8% in Eastern Europe and central Asia [17], 19% in East Asia [18] and 37% in the Middle East and North Africa [19]). This lack of progress has been associated with insufficient resources, inadequate coverage of women with antiretroviral treatment and HIV testing programmes not reaching the population groups at high risk for HIV infection. While sexual behaviour has changed to become safer in some countries and populations, sexual risk taking has increased in other settings. This is the case in most high-income countries in North America, western and central Europe and Australasia, where MSM are central to local HIV epidemics. In these countries, both high-risk sexual practices among MSM and HIV infections have been on the rise [20,21]. Condom use has increased in some countries, but declined in others. Proven effective interventions (e.g., prevention of mother-to-child transmission (PMTCT) and needle- and syringe-exchange programmes) have not achieved sufficiently high coverage in many countries [1].

Although trends in risky sexual practices have been linked to the trends in HIV incidence [22] and population-level behaviour change to the reduction in HIV prevalence, there are still challenges in linking behaviour-change programmes to specific HIV outcomes on the population level [2]. While new expectations have been raised about the role of antiretrovirals for HIV prevention, mixed progress was observed in access to cART, and only 61% of people eligible for treatment under the 2010 WHO guidelines received it (this is as little as 34% under the 2013 WHO guidelines). The Political Declaration has for the first time named and acknowledged the importance of such population groups as MSM, people who inject drugs and sex workers for HIV prevention, but in many settings, stigma and access to treatment and prevention services for these groups are still important challenges. Many low- and middle-income countries have stepped up their local investments in HIV prevention [2], but, regrettably, the lack of resources has remained a major issue: only US$18.9 billion was available from all sources for the AIDS response in 2012, and this was estimated to be 16–26% short of annual need [1].

It is at this time of some successes in HIV prevention and challenges in how to optimize the available resources and tools that the aspirational Political Declaration of commitment to fight the pandemic is necessary. This year's International AIDS Day marks the midpoint towards the deadline set by the Political Declaration in 2011. It is an opportunity for governments and each of us to revisit and reinvigorate the universal commitment to bring HIV infections to zero. Like never before, we have good cause to expect the next generation to be AIDS-free and new HIV infections to move towards zero. In the face of the 75 million people who have suffered from HIV/AIDS and the many more affected, the international community should keep the promise and bring this HIV pandemic to an end.
